# Efficient Rutin and Quercetin Biosynthesis through Flavonoids-Related Gene Expression in *Fagopyrum tataricum* Gaertn. Hairy Root Cultures with UV-B Irradiation

**DOI:** 10.3389/fpls.2016.00063

**Published:** 2016-02-04

**Authors:** Xuan Huang, Jingwen Yao, Yangyang Zhao, Dengfeng Xie, Xue Jiang, Ziqin Xu

**Affiliations:** Provincial Key Laboratory of Biotechnology of Shaanxi, Key Laboratory of Resource Biology and Biotechnology in Western China, Ministry of Education, College of Life Science, Northwest UniversityXi’an, China

**Keywords:** tartary buckwheat, genetic transformation, hairy root, rutin, UV-B, flavonoid biosynthetic genes

## Abstract

Transformed hairy roots had been efficiently induced from the seedlings of *Fagopyrum tataricum* Gaertn. due to the infection of *Agrobacterium rhizogenes*. Hairy roots were able to display active elongation with high root branching in 1/2 MS medium without growth regulators. The stable introduction of *rol*B and *aux*1 genes of *A. rhizogenes* WT strain 15834 into *F. tataricum* plants was confirmed by PCR analysis. Besides, the absence of *vir*D gene confirmed hairy root was bacteria-free. After six different media and different sources of concentration were tested, the culturing of TB7 hairy root line in 1/2 MS liquid medium supplemented with 30 g l^-1^ sucrose for 20 days resulted in a maximal biomass accumulation (13.5 g l^-1^ fresh weight, 1.78 g l^-1^ dry weight) and rutin content (0.85 mg g^-1^). The suspension culture of hairy roots led to a 45-fold biomass increase and a 4.11-fold rutin content increase in comparison with the suspension culture of non-transformed roots. The transformation frequency was enhanced through preculturing for 2 days followed by infection for 20 min. The UV-B stress treatment of hairy roots resulted in a striking increase of rutin and quercetin production. Furthermore, the hairy root lines of TB3, TB7, and TB28 were chosen to study the specific effects of UV-B on flavonoid accumulation and flavonoid biosynthetic gene expression by qRT-PCR. This study has demonstrated that the UV-B radiation was an effective elicitor that dramatically changed in the transcript abundance of *ftpAL*, *FtCHI*, *FtCHS*, *FtF3H*, and *FtFLS-1* in *F. tataricum* hairy roots.

## Introduction

As a significant food and medicinal species, tartary buckwheat (*Fagopyrum tataricum* Gaertn.; family Polygonaceae) is grown and used in the mountainous regions of Southwest China (Sichuan province), Northern India, Bhutan, and Nepal (Fabjan et al., 2003). The plant contains numerous functional components, including flavonoids, phenolic compounds, phytosterols, fagopyrins, d-chiro-inositol, and thiamin-binding proteins, which play essential role in antioxidant, hypocholesterolemic, and antidiabetic effects (Krkošková and Mrázová, 2005; Jiang et al., 2007; Tomotake et al., 2007; Yang and Ren, 2008; Yao et al., 2008; Qin et al., 2013).

The major functional components of *F. tartaricum*, such as rutin, quercetin, orientin, vitexin and kaempferol, had been demonstrated to be flavonoids In comparison with *Fagopyrum esculentum* (common buckwheat), as a source of dietary rutin and quercetin, *F. tartaricum* had higher contents of flavonoids and other phenolic compounds. As a secondary plant metabolite, rutin blocked the increase of capillary fragility related to hemorrhagic disease, reduced high blood pressure (Abeywardena and Head, 2001), decreased blood vessel permeability (with consequent antiedemic effect), lowered the risk of arteriosclerosis (Wojcicki et al., 1995), and displayed antioxidant activity (Watanabe, 1998; Park et al., 2000; Holasova et al., 2001; Krkošková and Mrázová, 2005). In comparison with common buckwheat, rutin content in tartary buckwheat was ∼3.2-fold higher in flowers, ∼3.1-fold higher in stems and ∼65-fold higher in seeds (Park et al., 2004). There had been increasing researches focusing on tartary buckwheat in recent years due to its remarkable health benefits associated with health.

Flavonoids are a class of secondary metabolites in plants involved in a great number of significant functions. They constitute a relatively diverse group of aromatic compounds derived from phenylalanine and malonyl-coenzyme. Phenylalanine ammonia lyase (PAL) catalyzes the conversion of phenylalanine to cinnamate. Based on this, *trans*-cinnamate is hydroxylated by cinnamic-4-hydroxylases (C4H) and is finally activated by the 4-coumarate/cinnamate coenzyme and 4-coumaryl-CoA-ligase (4CL), for the condensation of malonyl-CoA. As the major intermediates of flavonoid biosynthetic pathways chalcones are produced by the condensation of three molecules of malonyl-CoA and a single molecule of 4-coumaryl-CoA. The condensation of 4-coumaroyl-CoA and malonyl-CoA is conducted by chalcone synthase (CHS) to form either tetrahydroxy chalcone or trihydroxy chalcone. Chalcones are converted to the (2S)-flavanone naringenin by chalcone isomerases (CHIs) in a ring-closing step that forms the heterocyclic C-ring. From these central intermediates, the pathway diverges into several side branches for the synthesis of various classes of flavonoid molecules that are produced through the combined actions of functionalizing enzymes that could hydroxylate, reduce, alkylate, oxidize, and glycosylate the phenylpropanoid core structure (Kaneko et al., 2003; Fowler and Koffas, 2009; Santos et al., 2011); Flavanone 3-hydroxylase (F3H) catalyzes the stereospecific 3-hydroxylation of (2S)-flavanones to dihydroflavonols. Furthermore, dihydroflavonols are converted to flavonols and their glycosides through the corresponding flavonoid 3′-hydroxylase (F3′H) and flavonol synthase (FLS). These intermediates are further modified by varieties of hydroxylases, methyltransferases, reductases, and glycosyltransferases to form diverse flavonoids (e.g., quercetin and rutin) and isoflavonoids. For the biosynthesis of anthocyanins, dihydroflavonol reductase (DFR) catalyzes the stereospecific conversion of dihydroflavonols into the respective flavan-3,4-diols (leucoanthocyanins) through NADPH-dependent reduction at the 4-carbonyl. The leucoanthocyanins are further converted to the anthocyanidins by anthocyanidin synthase (ANS; Winkel-Shirley, 2001; Pandey and Sohng, 2013).

Hairy root cultures, established by the transformation of plants with *Agrobacterium rhizogenes*, typically showed an increased production of secondary metabolites. They were genetically and biochemically stable at rapid growth rate. Besides, they could synthesize useful natural compounds at the levels comparable to those of wild-type (WT) roots (Guillon et al., 2006a,b). Hairy root cultures of many plant species have already been widely studied regarding the production of secondary metabolites which could be used as pharmaceuticals, cosmetics, and food additives (Crane et al., 2006; Georgiev et al., 2007; Thiruvengadam et al., 2014). Biotechnological approaches which used hairy root culture have greatly enhanced the production of rutin by common buckwheat (Lee et al., 2007; Kim et al., 2010) and the production of phenolic compounds by tartary buckwheat (Kim et al., 2009; Thwe et al., 2013).

UV-B radiation has already been proved to be an efficient biotic stress to stimulate secondary metabolite accumulation in plant cell and tissue culture (Binder et al., 2009; Hao et al., 2009). According to previous work, the flavonoids of *F. esculentum* sprouts were produced as protective substances against the UV-B radiation (Ožbolt et al., 2008; Tsurunaga et al., 2013). To the best of our knowledge, there was no previous report about the effect of UV-B on functional metabolites accumulation in the hairy root culture of *F. tartaricum*.

To elucidate the role of UV-B as an abiotic elicitor in regulating synthesis and a yield of flavonoids and other secondary metabolites, an efficient protocol is needed for stable genetic transformation of hairy roots of *F. tartaricum*. Therefore, we established such a protocol and applied it into the study on induced biosynthesis and accumulation of flavonoids in hairy roots of *F. tartaricum*. Furthermore, we carried out a research program in order to investigate the effects of UV-B light and an addition of various concentrations of sucrose to liquid culture medium so as to enhance the flavonoids production in this study. Moreover, the expression of flavonoid biosynthetic genes was examined through quantitative real time PCR in combination with the change of flavonoids content, in order to analyze the relation between genes expression and metabolic biosynthesis of flavonoids.

## Materials and Methods

### Plant Material and Cultivation

Dehulled seeds of *F. tataricum* G. were surface-sterilized with 70% (v/v) ethanol for 1 min and 0.1% (v/v) mercuric chloride for 10 min, and then rinsed for four times in sterilized water. The treated seeds were sowed onto 1/2 MS medium (Murashige and Skoog, 1962) and solidified with 0.8% (w/v) agar. Before agar addition, the medium was adjusted to pH 5.8 and then sterilized through autoclaving at 121°C for 20 min.

Germinating seeds cultured the temperature of 25 ± 2°C in a growth chamber under a 16-h photoperiod with the flux rate of 35 μmol s^-1^ m^-2^. After 7 days, the hypocotyls and cotyledons of seedlings were cut into 0.5 cm × 0.5 cm pieces on a clean bench, and then transferred into Petri dishes, each of which contained 20 ml MS medium. The cut explants were cultured under the same conditions for 1–3 days as preculture before the inoculation.

Wild tartary buckwheat was planted in a test field at Northwest University (Xi’an, China) during the summers of year 2012 and 2013.

### Preparation of *Agrobacterium rhizogenes*

Cultured *A. rhizogenes* WT strain 15834 was utilized for hairy root induction. The bacteria were started from glycerol stock and grown at 28°C on 1.5% (w/v) of agar solidified YEB medium (Van Larebeke et al., 1977) with 250 mg/l penicillin for one night. Single colonies were grown at 28°C with shaking (180 rpm) in 20 ml YEB liquid medium with 250 mg/L penicillin for selection. *A. rhizogenes* suspension culture was kept overnight until reaching the density of OD_600_ = 0.5. Cells were collected by centrifugation (4000 rpm, 5 min) and were resuspended in 1/2 MS liquid medium with supplement 30 g/L sucrose and acetosyringone 200 mmol/L. Cell suspensions at density OD_600_ = 0.6 were used for inoculation.

### *A. rhizogenes* -Mediated Transformation

Hypocotyls and cotyledons from 7-days-old plants were cut into ∼0.5 cm pieces. Excised explants were dipped into *A. rhizogenes* 15834 suspensions in liquid inoculation medium for 10, 12, 15, or 20 min, blotted dry on sterile filter paper, and incubated in the dark at 25°C on the agar-solidified MS medium. As a control, a few explants were placed in 1/2 MS liquid medium and were cultured in the same way. After 1∼3 days of co-culture, explants were transferred onto solidified MS medium supplemented with 500 mg l^-1^ cefotaxime sodium. Explants that produced hairy roots (usually within 2 weeks after infection) were selected for further study. Roots (length 1.5–2.0 cm) that developed on the explants were excised aseptically, transferred onto MS medium supplemented with 400 mg l^-1^ cefotaxime in 9-cm Petri dishes, and incubated under the conditions described in Section “Plant Material and Cultivation.” Roots were grown for 14 days. Furthermore, 0.3 g fresh weight (FW) was transferred into 250-ml Erlenmeyer flasks containing 50 ml MS, 1/2 MS, N6, ½ N6, B5 and 1/2 B5 liquid medium without growth regulators. Cultures were incubated on a shaker (100 rpm) as above, and roots were subcultured in every 14 days. Cefotaxime concentration was gradually reduced to zero in the MS liquid medium. Roots were kept at 25 ± 2°C under standard cool white fluorescent tubes with the flux rate of 35 μmol s^-1^ m^-2^ and a 16-h photoperiod. Experiments were conducted in duplicate with three flasks per culture condition. As hairy roots can grew very well on medium without growth regulators, normal roots could hardly grow on the same medium. Therefore, as a control, roots excised from *in vitro* germinated seedlings were cultured in MS liquid medium without growth regulators. Meanwhile, transformation efficiency was calculated with each treatment. The transformation efficiency equaled to the number of explants inducing hairy roots/total number of explants × 100%.

### Genomic DNA Extraction and PCR Analysis

Genomic DNA was extracted from hairy roots and WT (seedling grew in 1/2 MS medium as control) roots of *F. tataricum* by the CTAB procedure (Doyle and Doyle, 1990). The Ri-plasmid of *A. rhizogenes* was extracted from strain 15834 by the SDS/alkaline lysis method (Petit et al., 1983), being used as a positive control. Integration of T-DNA responsible for hairy root formation was confirmed by PCR analysis using *rol*B, *aux*1, and *Vir*D specific primers. The sequences of primers used in the experiment were for *rolB* (Forward: 5′-GAT ATA TGC CAA ATT TAC ACT AG-3′; Reverse: 5′-GTT AAC AAA GTA GGA AAC AGG-3′, the expected PCR product was 564 bp), *aux*1 (Forward: 5′-TTC GAA GGA AGC TTG TCA GAA-3′; Reverse: 5′-CTT AAA TCC GTG TGA CCA TAG-3′, the expected PCR product was 350 bp) and *Vir*D (Forward: 5′-ATG TCG CAA GGC AGT AAG CCC A-3′; Reverse: 5′-GCA GTC TTT CAG CAG GAC GAG CAA-3′, the expected PCR product was 438 bp). The PCR mixture consisted of 5 μl 10× PCR buffer (Takara Biotech; Japan), 2.5 μl of 100 nM dNTPs (Takara), 1 μl primer, 1 μl Taq polymerase (Takara), and 1 μl plant genomic DNA (or 1 μl plasmid DNA), in a final volume of 50 μl. The amplification conditions were: predenature for 5 min at 94°C; denature for 1 min at 94°C; anneal primer for 55 s at 52°C (*rol*B)/ anneal primer for 55 s at 55°C (*aux*1)/ anneal primer for 55 s at 56°C (*Vir*D); extension for 1 min at 72°C; repeat for 30 cycles; and final extension at 72°C for 10 min. PCR results were checked by agarose gel electrophoresis (100 v/h) with DL5000 ladder marker (Takara), detected by ethidium bromide staining, and photographed by a gel documentation system (Bio-Rad; Hercules, CA, USA).

### Total RNA Extraction and Expression Analysis of Flavonoid Biosythetic Genes by qRT-PCR

Total RNA was isolated from *F. tataricum* wild and transgenic hairy roots by utilizing the RNeasy Plant Mini Kit (Qiagen; Valencia, CA, USA). The RNA integrity was checked by 1.2% ethidium bromide stained RNA gel through the absorbance spectrum at 260: 280 nm wavelength by NanoVue Plus Spectrophotometer (GE Healthcare Bio-Science Crop., USA). The cDNA was synthesized from 1 μg of DNA free total RNA and reverse transcribed utilizing PrimeScript^®^ 1st Strand cDNA Synthesis Kit (Takara). The resulting cDNA products were used as the template for real time-PCR analysis.

Quantitative real-time PCR was performed for the transcriptional level analysis of flavonoid biosynthesis genes in a BIO-RAD CFX96 Real-time PCR system (Bio-Rad Laboratories, Hercules, CA, USA). The gene-specific primer sets were designed as previous information described by Li et al. (2010). Real-time PCR was carried out in a 20 μl reaction volume including 0.5 μl of each primer, 5 μl of template cDNA and 10 μl of SYBR Green (SYBR^®^ *premix Ex Taq*^TM^, Takara). According to Thwe et al. (2013), the program was executed. The histone H3 gene was used as reference gene (Timotijevic et al., 2010). Fluorescent intensity data were acquired during the extension step. The transcript levels were checked through utilizing a standard curve. Identical PCR conditions were used for all targets. The significant differences between cultivars were evaluated from three replicates of each sample.

### Measurement of Flavonoid Content (Rutin and Quercetin) by High-Performance Liquid Chromatography (HPLC)

Harvested hairy roots (1 g) were frozen in liquid N_2_, ground to a fine powder with a mortar and pestle, and extracted twice in methanol (50 ml) for 24 h at 5°C. Extracts were vacuum-dried at 80°C and dissolved in 10 ml methanol. The solution was filtered through a poly filter (pore size 0.45 μm) and diluted twofold with methanol. Extracts were used to analyze the rutin and quercetin by HPLC (Waters 2695 Sespartions Module and Waters 2996 Photodiode Array Detector) on a C18-column (Hypersil ODS, 250 mm × 4.6 mm) at 30°C. The mobile phase consisted of methanol (solvent A) and 1% (v/v) glacial acetic acid (solvent B) with the flow rate of 1 mL min^-1^. The solvent gradient was from 40% solvent A/60% solvent B to 65% solvent A/35% solvent B over 35 min. Sample (rutin and quercetin) detection wavelength were 257 and 370 nm, respectively; injection volume equaled to 20 μl. The rutin and quercetin were detected and quantified with the authentic standards obtained from the Institute for Identification of Pharmaceutical and Biological Products (Beijing, China), which identified by the comparison with retention times and spectral characteristics of authentic standards. The quantitative values were calculated from the calibration curves (**Supplementary Material, S1** and **S2**). All samples were run in triplicate (A HPLC chromatogram of hairy root line is shown in **Supplementary Material, S3**).

### Growth Kinetics of Cultured Transformed Roots

Hairy roots (0.3 g FW) were inoculated in a 250-ml Erlenmeyer flasks containing 50 ml basal liquid medium supplemented with sucrose. Biomass accumulation and flavonoids production were optimized through evaluating growth kinetics at various time intervals (4, 8, 12, 16, 20, 24 days). The media (MS, 1/2 MS, N6, 1/2 N6, B5, and 1/2 B5) and various concentrations of sucrose (10, 20, 30, 40, 50 g l^-1^) were evaluated to maximize the root biomass growth. Flasks were cultured with shaking (100 rpm) at 25 ± 2°C with the flux rate of 35 μmol s^-1^ m^-2^ and a 16-h photoperiod. Hairy root biomass (FW and DW) and flavonoids production were measured during the 24 days of culture.

### UV-B Light Stress Treatment

After hairy roots had grown to 2 g (FW) in 1/2 MS liquid medium, they were exposed to UV light (UV-B 313 lamps; Q-Panel; Cleveland, OH, USA) for 30 min. The lamps were wrapped in cellulose diacetate filters to block the UV-C range (wavelengths < 280 nm). The maximal radiation peak was at wavelength 302 nm. The UV-B light intensity on the sample surface was 1.26 μW/cm^2^, and the total energy supply was 0.34 J/cm^2^. The treatment continued for 3 days and the experiments of UV-B treatment were repeated at least three times. Both of rutin and quercetin contents were analyzed as in Section “Measurement of Flavonoid Content (Rutin and Quercetin) by High-Performance Liquid Chromatography (HPLC).” Non-transformed roots and wild plants were subjected to UV-B light stress as controls. All parts of the wild plants were examined by HPLC. Meanwhile, the expression of flavoniod biosynthesis genes was detected after the hairy roots were treated by UV-B irradiation. The changes of transcription abundance were analyzed by qRT-PCR as shown in Section “Total RNA Extraction and Expression Analysis of Flavonoid Biosythetic Genes by qRT-PCR.”

### Statistical Analysis

All data analyses were performed using the Origin software program, V. 8.0. Values were expressed as mean ± SE. One-way ANOVA was applied in statistical analysis. Differences between means were evaluated by Duncan’s multiple range test, and were considered to be significant for *P* < 0.05.

## Results

### Establishment of Hairy Root Induction

The establishment of an efficient and reliable transformation system was proved to be highly desirable for the whole culture process of hairy root. Base on the preliminary experiments, explants age and co-cultivation time were investigated systematically in this work. Finally, 168 explants of *F. tatarium* were induced to hairy roots in 847 explants, and the transformation frequency was 19.83%.

#### Effects of Preculture Time on Transformation Frequency of *F. tatarium*

**FIGURE 1:**
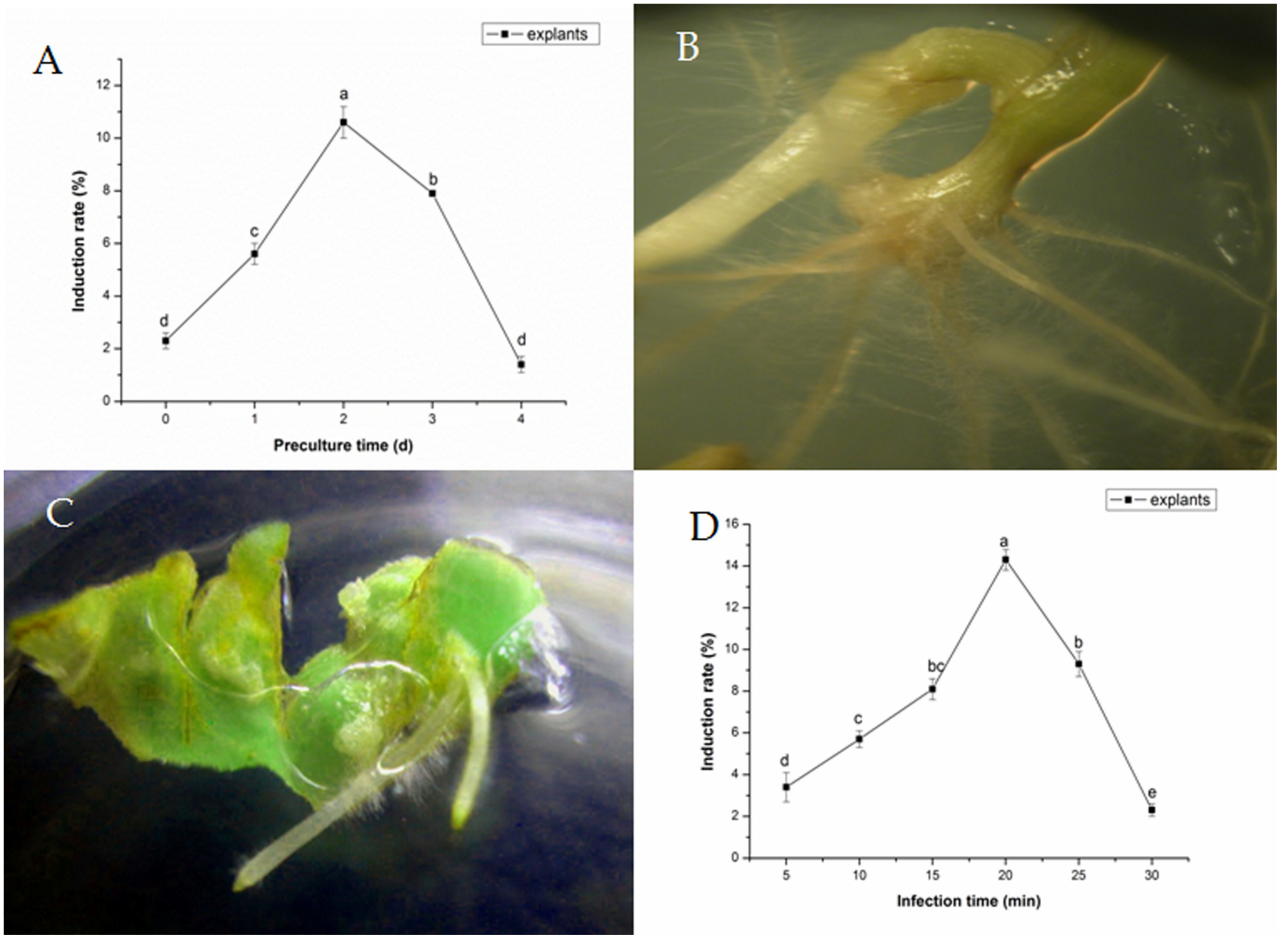
**Effects of preculture time **(A)** and *Agrobacterium rhizogenes* infection time.** **(B)** Development of hairy roots from a petiole segment within 10 days. **(C)** Development of hairy roots from cotyledon within 5–7 days. **(D)** On transformation frequency of *Fagopyrum tataricum.*

Transformation frequency varied based on different preculture time (**Figure 1A**). Explants became more sensitive to the integration of T-DNA as preculture time increased. Transformation frequencies of explants were improved by 1–2 days preculture, in comparison with controls, reaching the maximum degree after 2 days of preculture. Besides, the induce rate could reach to 10.6 ± 0.6%. At longer preculture times, transformation frequencies decreased rapidly. Five to twelve hairy roots per stem were induced from hypocotyl cut ends or petiole within 2 weeks (**Figure 1B**). Meanwhile, no hairy roots were observed on explant without infection. Two to five hairy roots per explant were induced from cotyledons of tartary buckwheat with *A. rhizogenes* strain 15834 (**Figure 1C**).

#### Effect of *A. rhizogenes* Infection Time on Transformation Frequency

Subjected to various transformation times, excised explants were dipped into *A. rhizogenes* in liquid inoculation medium. The optimal transformation times were about 20 min for explants, and the induce rate was 14.3 ± 0.5% (**Figure 1D**); All these resulted in greater transformation efficiency and less damages to the explants.

### Confirmation of Genetic Transformation of Hairy Roots by PCR

**FIGURE 2:**
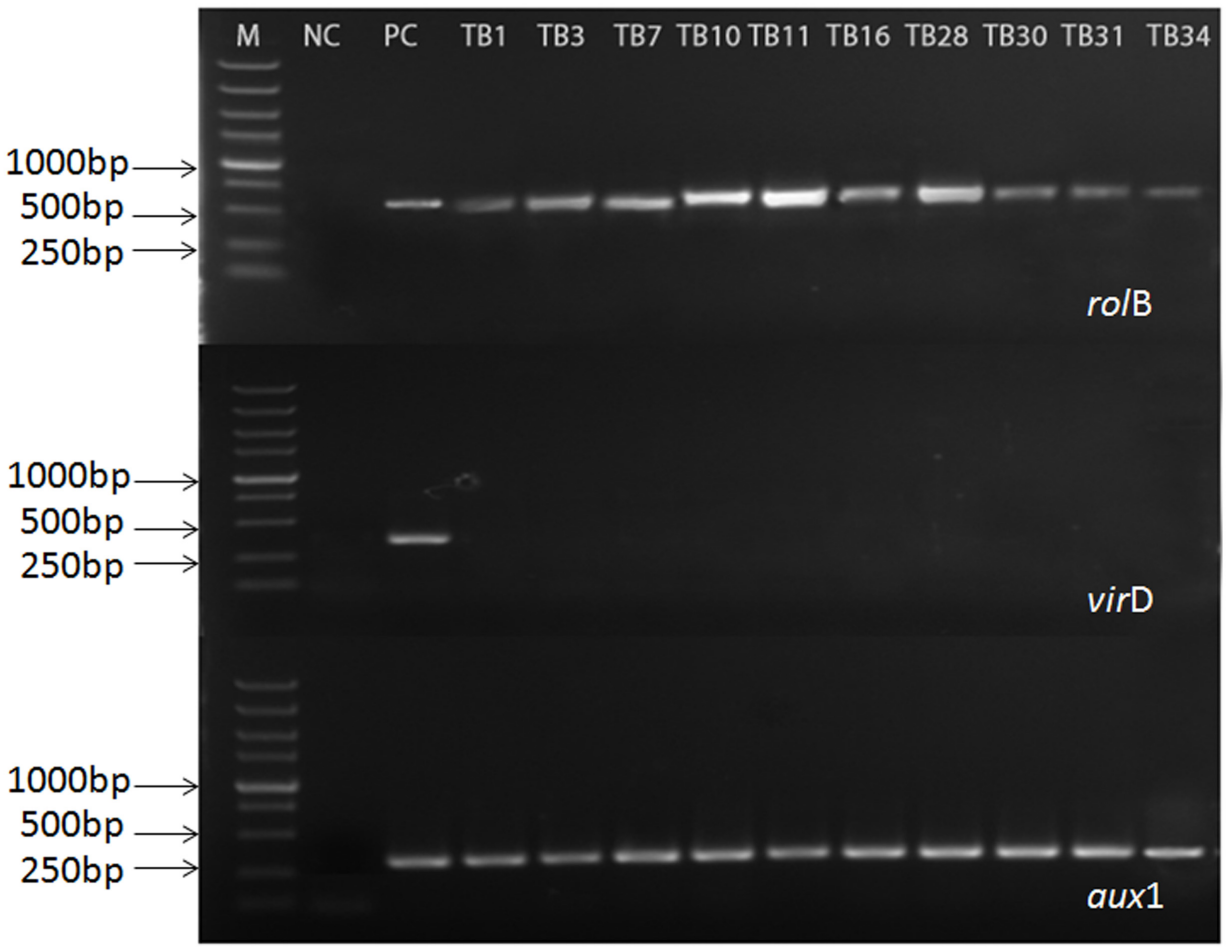
**Polymerase chain reaction (PCR) analysis for the presence of *rolB*, *aux1*, and *virD* genes in hairy root lines of *F. tataricum* transformed by *A. rhizogenes* strain 15834.** Lane M, marker. Lane 1, non-transformed roots [negative control (NC)]; Lane 2, plasmid DNA [positive control (PC)]; Lanes 4–13, transformed hairy root clones.

In order to evaluate the genetic status of the selected hairy root, the PCR-based analysis was performed on the targeted *rol*B, *aux*1, and *vir*D genes. The *rol*B gene (located at the pRi TL-DNA segment) and the *aux*1 gene (located at the pRi TR-DNA segment), were diagnostic for T-DNA integration into the host genome of hairy root. The *Vir*D gene (located outside the pRi T-DNA segment) was used to check the presence of any remaining *Agrobacteria* in hairy root of *F. tatarium.* Furthermore, the root of aseptic plantlets and *A. rhizogenes* 15834 Ri plasmid were used as negative and positive controls respectively. According to the part results shown in **Figure 2**, the coexistence of *rol*B and *aux*1 genes indicated that the established hairy root lines integrated the pRi T-DNA of *A. rhizogenes* 15834 successfully. Furthermore, the absence of *vir*D gene confirmed that all those hairy roots were bacteria-free (The rest of results are shown in **Supplementary Material, S4**).

From the entire obtained 356 hairy root lines, we chose 60 lines which grew quickly in medium without plant growth regulators to the processing of PCR analysis. The presence of both of *rol*B and *aux*1 genes and the absence of *vir*D gene were observed in 20 hairy root lines. The results showed that these cultures had no *Agrobacterium* contamination, and that both of T-DNA fragments were integrated into the genome of these root lines.

### Effects of Various Media and Sucrose Concentration on Biomass Accumulation

**FIGURE 3:**
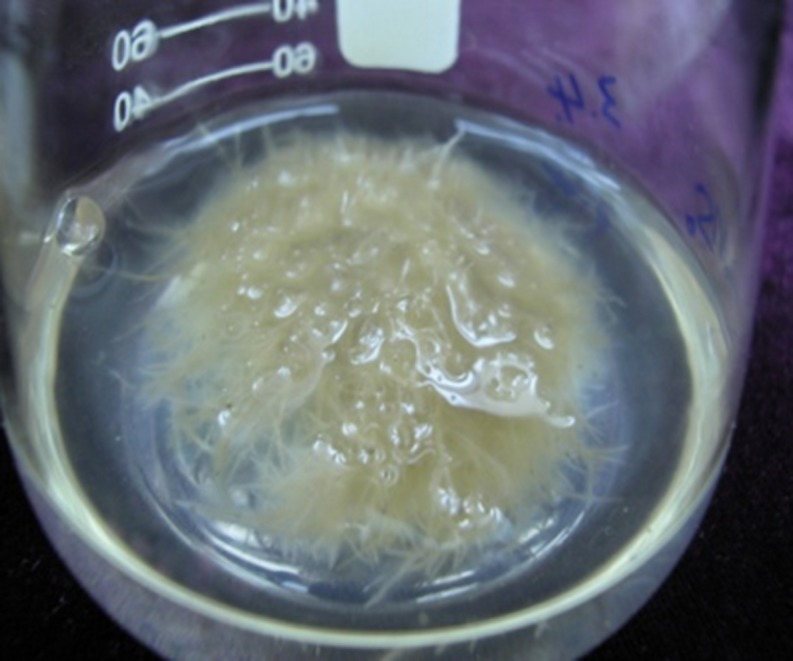
**Rapidly growing hairy root line TB7 culture in liquid MS medium without growth regulators after 3 weeks**.

We used several media (MS, 1/2 MS, B5, 1/2 B5, N6, 1/2 N6) for hairy root culture (**Table 1**). Hairy roots (TB7 line) were cultured for 20 days in six media for observation and for accurate measurement of FW and DW. Amongst the six media, 1/2 MS was the best for biomass accumulation (**Table 1**). Hairy roots were cultured in 1/2 MS liquid medium and presented the most rapid proliferation (**Figure 3**). The highest values of biomass accumulation (13.5 g l^-1^ FW; 1.78 g l^-1^ DW) were recorded for 1/2 MS medium, in which a thick mass grew in the bottle within 20 days. The growth cycles were generally similar in 1/2 MS solid vs. liquid media. However, the hairy root growth was slower in the solid medium. The condition of biomass accumulation in B5 and N6 media (including 1/2 B5 and 1/2 N6) was similar, which FW and DW of biomass were ∼50% lower than in 1/2 MS liquid medium, and the rutin content was lower than in 1/2 MS liquid medium.

**Table 1:** Effects of different media and sucrose concentrations on biomass production of hairy root cultures in 1/2 MS medium.

	Fresh weight (g)	Dry weight (g)
**Medium**		
MS	10.42 ± 0.68^b^	1.23 ± 0.21^b^	
1/2 MS	13.50 ± 0.60^a^	1.78 ± 0.15^a^	
B5	5.64 ± 0.73^d^	0.86 ± 0.24^d^	
1/2 B5	6.74 ± 0.70^c^	0.82 ± 0.19^c^	
N6	5.01 ± 0.89^d^	0.45 ± 0.17^d^	
1/2 N6	6.24 ± 0.96^c^	0.56 ± 0.24^c^	
**Sucrose concentration (g/l)**			
10	5.56 ± 0.32^d^	0.58 ± 0.34^d^	
20	11.65 ± 1.2^b^	1.28 ± 0.50^b^	
30	13.50 ± 0.60^a^	1.78 ± 0.15^a^	
40	12.11 ± 0.22^b^	1.19 ± 0.16^b^	
50	8.34 ± 0.45^c^	0.81 ± 0.71^c^	


*Roots (0.3 g FW per flask) were cultured for 20 days in 250-ml Erlenmeyer flasks containing 50 ml medium for 20 days. Data shown are mean ± SE of three replicates. Each experiment was performed in triplicate. Means with common letters are not significantly different at *P* < 0.05 according to Duncan’s multiple range test.*

As hairy roots were grew very well in 1/2 MS and MS media, we established various concentrations of sucrose (10–50 g l^-1^) in 1/2 MS medium, and examined their effects on hairy root growth. As the concentration of sucrose increased, hairy roots changed from white to brown, and became slower-growing. The growth of hairy root reached its maximum in 30 g l^-1^ sucrose, and decreased greatly at the concentrations higher or lower than this value.

### Kinetics of *F. tataricum* Hairy Root Growth and Flavonoids Accumulation

**FIGURE 4:**
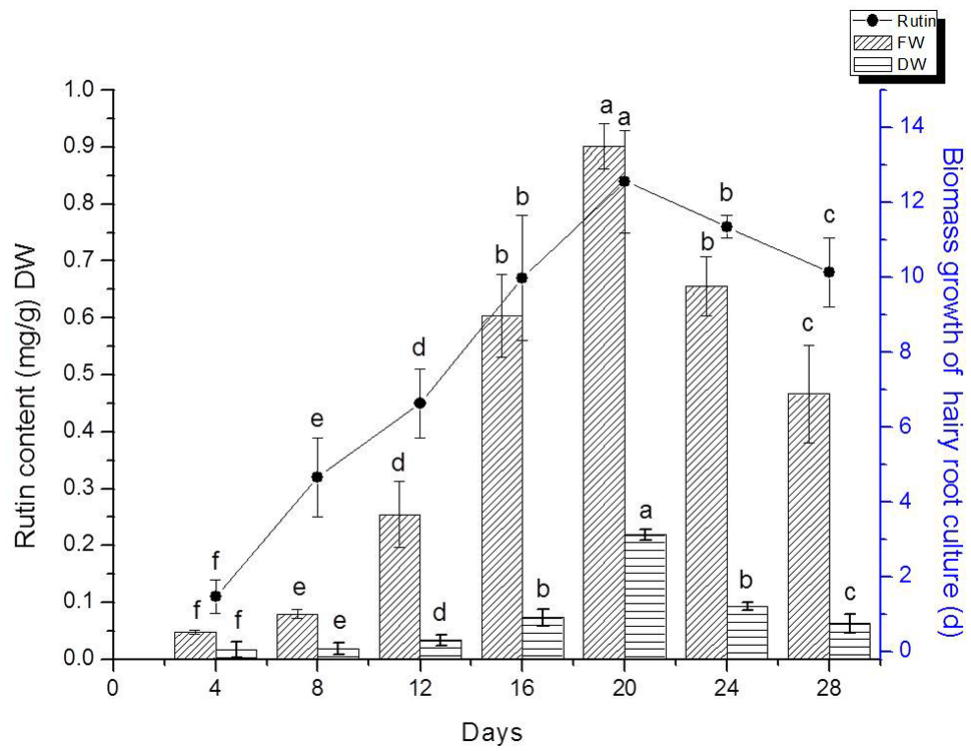
**Hairy root line TB7 growth as a function of time, and kinetics of rutin production.** Points represent mean ± SE of three replicates. Each experiment was performed in triplicate. Means with common letters are not significantly different at *P* < 0.05 according to Duncan’s multiple range test.

We detected flavonoids yield of 20 lines of hairy root which had been confirmed by PCR analysis. The result indicated that line TB7 had the highest content of rutin and quercetin (Data isn’t shown). Therefore, TB7 were chosen to investigate the kinetics studies on biomass growth and flavonids accumulation in hairy root cultures of *F. tataricum* as shown in **Figure 4**. Hairy roots (initial FW 0.3 g) were cultured for 30 days in 1/2 MS medium containing 30 g l^-1^ sucrose. Root growth occurred primarily during the first 4–5 days, and then leveled off during days 20–24. Hairy roots were initially white or pale yellow, yet became brown and slow-growing after day 24, and required so subculturing in every 24 days. The maximal rutin yield was obtained (0.85 mg g^-1^) when the hairy root cultures were cultured on days 20. At the same time, the maximal FW and DW were 13.5 and 1.78 g, respectively. Both of biomass and rutin contents declined rapidly after 20 days.

### Effect of UV-B Light Stress Treatment on Flavonoids Production

We evaluated the resistance of wild tartary buckwheat plants and hairy roots to the UV-B light stress, in regard to possible enhancement of rutin and quercetin production (**Table 2**). TB7 hairy root lines were chosen to study the specific effects of UV-B on flavonoid accumulation. The rutin and quercetin content of stems, leaves, flowers, and WT (non-transformed) roots were compared with that of hairy roots. Following the UV-B stress treatment, the rutin content of hairy roots was strikingly higher than that in non-transformed roots. The increase in rutin content of treated hairy roots (from 0.93 to 4.82 mg g^-1^) was 5.18-fold higher than in WT roots. Hairy roots were more sensitive to UV-B stress treatment than to non-transformed roots, flowers, or stems. The relative order of rutin content increase under UV-B stress was leaves (9.35-fold) > hairy roots (5.18-fold) > stems (3.57-fold) > non-transformed roots (2.95-fold) > flowers (2.66-fold). Quercetin yield could not be detected in no-transformed root before the UV-B treatment. However, quercetin content was found in hairy root TB7 line, which increased drastically in hairy root with exposure due to UV-B (from 0.02 to 0.04 mg g^-1^). Meanwhile, quercetin was detected in leaves, flowers and stems of *F. tataricum*, and the yield was increased substantially after the treated UV-B irradiation.

**Table 2:** Effects of UV-B stress on rutin and queretin content of various plant parts.

	Rutin content (mg/g DW)		Quercetin content (mg/g DW)	
		
Sample	No treatment	UV-B irradiation	No treatment	UV-B irradiation
Transgenic hairy roots	0.93 ± 0.02^d^	4.82 ± 0.03^d^	0.02 ± 0.01^d^	0.04 ± 0.01^d^
Non-transformed roots	0.21 ± 0.01^e^	0.61 ± 0.03^e^	0	0.01 ± 0.01^e^
Leaves	3.19 ± 0.03^b^	29.79 ± 0.12^a^	0.19 ± 0.02^c^	1.26 ± 0.07^b^
Flowers	7.0961 ± 0.03^a^	19.0 ± 0.10^b^	0.71 ± 0.12^a^	1.32 ± 0.67^a^
Stems	1.03 ± 0.01^c^	3.45 ± 0.09^c^	0.22 ± 0.04^b^	0.67 ± 0.18^c^


*After exposed to UV-B light for 30 min for 3 days, rutin and quercetin content of hairy roots were analyzed by HPLC. Data shown are mean ± SE of three replicates. Each experiment was performed in triplicate. Means with different letters are significantly different at *P* < 0.05 according to Duncan’s multiple range test.*

### Expression of Flavonoid Biosynthetic Genes in Hairy Roots of *F. tataricum* with UV-B Irradiation

**FIGURE 5:**
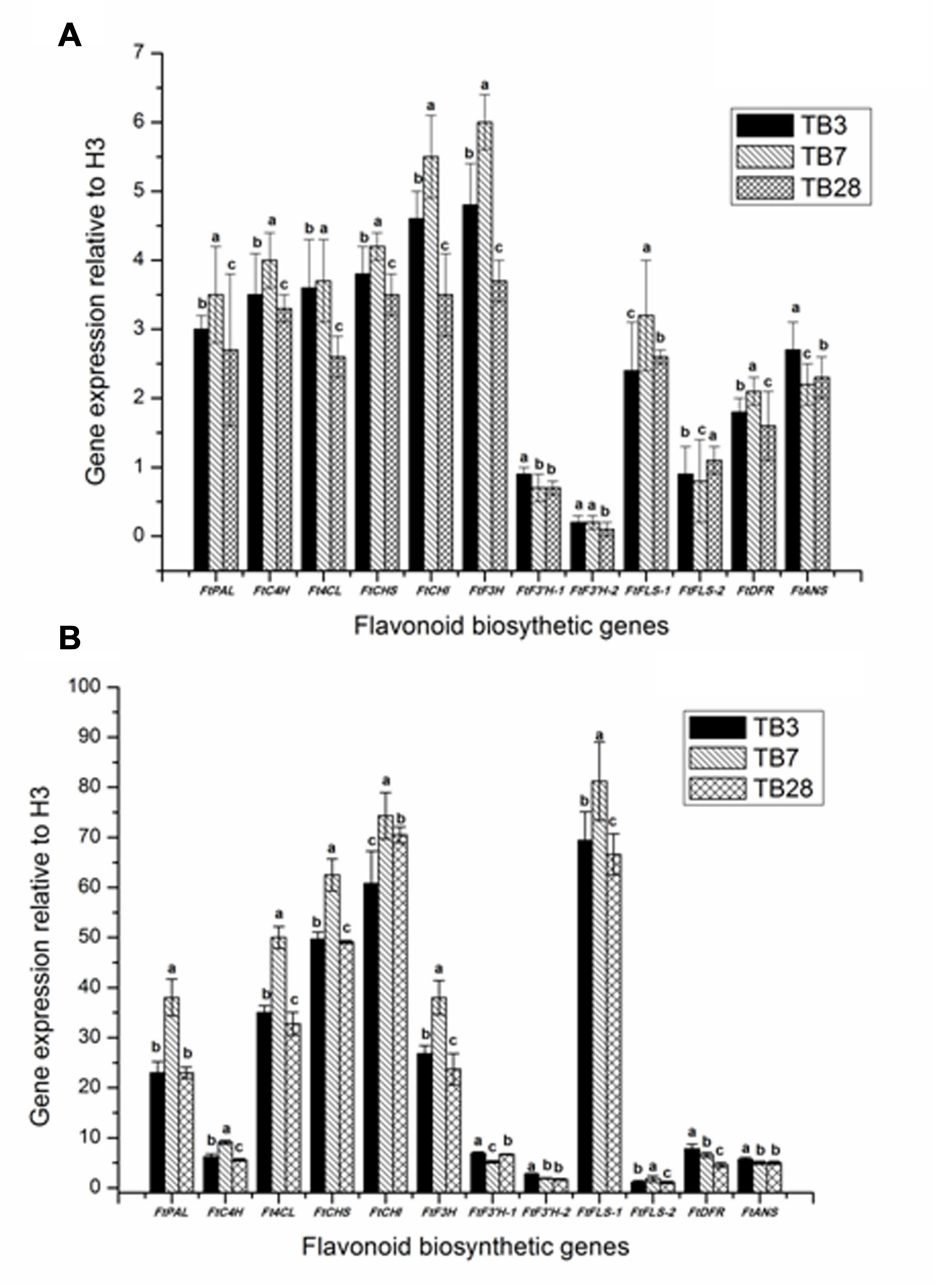
**The effects of UV radiation on transcript abundance of flavonoid biosynthetic genes.** Data shown are the average mean ± SE of three replicates (*n* = 3). Different letters indicate statistical significane (*P* < 0.05) according to one-way ANOVA and Duncan’s multiple range test. **(A)** The expression of flavonoid biosynthetic genes in hairy root lines TB3, TB7, and TB28 of *F. tataricum*; **(B)** the expression of flavonoid biosynthesis genes in hairy root lines TB3, TB7, and TB28 of *F. tataricum*. after UV-B treatment.

To investigate biosynthesis of flavonoid genes in *F. tataricum*, the expression levels of biosynthesis genes in the hairy roots of *F. tataricum* were examined by qRT-PCR (**Figure 5A**). The expression levels of *ftpAL*, *FtC4H*, *Ft4CL*, *FtCHS*, *FtCHI*, *FtF3H*, *FtF3*′*H-1*, *FtF3*′*H-2*, *FtFLS-1*, *FtFLS-2*, *FtDFR*, and *FtANS* were shown. Although the gene transcripts for all of these enzymes were expressed in hairy roots (TB3, TB7, and TB28 lines) of *F. tataricum*, the expression levels were upregulated in TB7 than TB3 and TB28 hairy root lines except in *FtF3*′*H-1*, *FtF3*′*H-2*, *FtFLS-2*, and *FtANS*. In particular expression levels of *ftpAL, FtC4H*, *FtCHI*, *FtF3H*, and *FtFLS-1* in TB7 hairy root were significantly higher than the expression levels of TB3 and TB28 lines.

In order to understand the role of UV-B in regulating flavonoid biosynthesis, the transcript abundance for genes were involved in the flavonoid biosynthetic pathway and were analyzed by qRT-PCR (**Figure 5B**). The hairy root lines of TB3, TB7, and TB28 were also chosen to study the specific effects of UV-B exposure on gene expression. Although the gene transcripts for all the genes had increased the expression in three hairy roots lines, the expression levels were upregulated in TB7 than in TB3 and TB28 hairy root lines except in *FtF3*′*H-1*, *FtF3*′*H-2*, *FtDFR*, and *FtANS*. The key regulate genes of flavonoid biosynthesis, including *FtpAL*, *FtCHS*, *FtCHI*, *FtF3H*, and *FtFtFLS-1*, were found to be responsive to UV-B exposure drastically. *FtFLS-1* showed the highest transcript abundance in the UV-B exposure treatment, which was 30–40 fold higher than of no UV-B treatment. A significant UV-B induced was also observed in the transcript abundance of *FtCHI and Ft CHS*, which was 20–30 fold higher than no UV-B treatment. In contrast, the transcript abundance of *FtF3*′*H-1*, *FtF3*′*H-2*, *FtFLS-2*, *FtDFR*, and *FtANS* were slightly enhanced to response to the UV-B exposure treatments.

As shown in **Figure 5**, genes of flavonoid biosynthesis in tartary buckwheat have been almost entirely elucidated.

## Discussion

*Agrobacterium rhizogenes* as a useful tool for gene transfer has been widely applied in many plant species. *A. rhizogenes* strain 15834 was often used to induce hairy root formation. Kim et al. (2010) obtained the hairy root of *F. esculentum* by *A. rhizogenes* 15834 successfully. *Taraxacum platycarpum* (Lee et al., 2004) and *Panax ginseng* (Yang and Choi, 2000) also underwent successful transformation with *A. rhizogenes* 15834. Meanwhile, Kim et al. (2009) and Park et al. (2011) reported that they obtained hairy root after inoculating sterile young stems of *F. tataricum* with *A. rhizogenes* strain R1000.

Transformation efficiency was affected by bacterial growth stage, infection time, pre-treatment of explant, as well as light and temperature conditions. Preculture of explants in MS medium for 2 days prior to transformation enhanced transformation efficiency. The optimal time of transformation was ∼20 min for explant. Plant tissues might be injured if the infection time was too long, and the infection time varied according to the plant species and type of explant. Chen et al. (2008) showed that longer preculture time might reduce explant viability, which resulted in injury. On the other hand, explants might wither more easily in the absence of a preculture process.

We obtained the maximal transformation frequency of *F. tataricum* by *Agrobacterium* only after suitable preculture. However, this conclusion might not be applied to all the plant species. Kim et al. (2004) used *Agrobacterium* to infect the *Perilla frutescens* explants, finding that the transformation frequency was much higher for non-precultured than for precultured explants.

The growth rate of hairy roots was greatly affected by the culture medium and various culture conditions. In present study, culturing in 1/2 MS liquid medium led to the most rapid proliferation of hairy roots, and biomass (FW) had increased ∼45-fold in 20 days. The growth of hairy root was exponential from days 0 to 20, and then entered into a stationary phase during days 20–25. These findings indicated that hairy root liquid cultures of tartary buckwheat were potentially useful for large-scale biomass production. Park et al. (2011) also reported that the dry weight of hairy root enhanced 25-fold in the MS liquid medium during 21 days. *Polygonum multiflorum* Thunb. hairy root showed that the biomass (initially 0.5 g FW) increased 9.5-fold after being cultured in hormone-free MS liquid medium for 20 days (Thiruvengadam et al., 2014).

As the major carbon source, sucrose was extremely essential for the *F. tataricum* hairy root growth. In the present study, hairy roots grew rapidly in high sucrose concentrations. The optimal sucrose concentration was found to be 30 g l^-1^, at which biomass accumulation and rutin content were maximal. The growth of hairy root was strikingly lower at sucrose concentrations above or below 30 g l^-1^. Higher concentrations also resulted in the alteration of root morphology (e.g., root calluses, inhibition of lateral branching) probably due to osmotic stress (Hamill et al., 1987). Yu et al. (1996) found that the sucrose level was affected hairy root production in *Solanum avidare*. The levels of secondary metabolites produced by *in vitro* cultures could vary dramatically. As a matter of fact, most previous studies were focusing on nutrient composition in medium to achieve the optimized accumulation of metabolites in cultured cells (Rao and Ravishankar, 2002).

Since the biosynthesis of many secondary metabolites in plant is usually considered as a common defense response of plants to biotic and abiotic stresses, their accumulation could be stimulated by biotic and abiotic elicitors (Zhao et al., 2014a). Therefore, elicitation, as treatment of plant tissue cultures with elicitors, is one of the most effective strategies to enhance secondary metabolites production in plant tissue cultures. The most common and effective elicitors used in previous studies mainly included heavy metal ions, UV radiation (abiotic), the component of microbial cells, especially poly- and oligosaccharides (biotic), and the signaling molecules in plant defense responses, such as salicylic acid (SA) and methyl jasmonate (MJ; Chen and Chen, 2000; Broeckling et al., 2005; Prakash and Srivastava, 2008; Smetanska, 2008; Ionkova, 2009; Zhao et al., 2010).

Flavonoids are produced as protective substances against UV-B radiation in plant. As an effective abiotic elicitor, some studies have described the production of flavonoids by buckwheat sprouts in response to UV-B irradiation (Kreft et al., 2002; Eguchi and Sato, 2009). Rutin (sometimes called vitamin P) displays strong antioxidant activity which could alleviate the damage from UV-B stress. Tsurunaga et al. (2013) found that rutin content and radical scavenging activity of buckwheat sprouts were enhanced under various levels of UV-B radiation. In the present study, rutin and quercetin content of hairy roots and all parts of tartary buckwheat were increased under UV-B stress. The maximal increase of rutin content (from 3.19 to 29.79 mg g^-1^, 9.35-fold) was observed in leaves. Interestingly, the next-highest increase of rutin content (from 0.93 to 4.82 mg g^-1^, 5.18-fold) was observed in hairy roots. This phenomenon might result from the insertion site of T-DNA during transformation; the underlying mechanism requires further investigation. In a previous study of buckwheat, Kim et al. (2010) found that rutin content was ∼2.4-fold higher in hairy roots than in WT roots. These findings are consistent with those of transformation studies on other plants, which suggested that *Agrobacterium* transfection might greatly enhance rutin content (Fu et al., 2006). According previous work, some work indicated that biotic elicitors can also enhance rutin and quercetin production in *F. tataricum* hairy root, e.g., Yeast polysaccharide (Zhao et al., 2014a) and exogenous fungal mycelia (Zhao et al., 2014b).

To understand the role of UV-B in regulating flavonoid biosynthesis, the transcript abundance for key enzymes genes involved in the flavonoid biosynthetic pathway were analyzed by qRT-PCR. Previous studies have extensively described the UV-B induction of those gene expressions (Frohnmeyer et al., 1992; Kubasek et al., 1992; Christie and Jenkins, 1996; Kliebenstein et al., 2002; Stracke et al., 2010). Flavonoid synthesis in plants is induced by perceiving the UV-B light with photoreceptors and by the expression of phenylpropanoid biosynthesis genes (Rizzini et al., 2011). These works considered the useage of flavonoid biosynthetic key enzymes PAL.C4H, CHI, CHS, FLC, F3H, etc. as an excellent UV-B signaling response marker. Amongst these genes, activated by the UV light, PAL played an important role in the first step of the flavonoid biosynthesis pathway. According to Hahlbrock et al. (2003), the promoter on PAL was in Box-P and that box-P-binding factor 1 was induced by UV stimulation and up-regulated the gene expression of PAL and followed the binding to Box-P. In the UV irradiation of rice seedlings, it has been reported that PAL increased at 12 h to its maximum expression and increases the contents of flavonoids (Jin et al., 2000). Hao et al. (2009) also reported that activation of PAL in *Ginkgo biloba* callus could be induced by UV-B, and flavonid biosynthesis could be stimulated. In *Arabidopsis*, the UV-B-mediate induction of *CHS* expression was UVR8-, COP1-, and HY-5-dependent with proteins belonging to the UV-B signaling pathway (Kliebenstein et al., 2002; Stracke et al., 2010). It has been widely known that HY5 can directly bind to the *CHS* promoter. However, this was not sufficient for the *CHS* transcriptional activation. However, the overexpression of HY5 fused with an activation domain was sufficient for the *CHS* expression induction, indicating that a presently unknown UV-B activated transcription factor must be involved as well (Stracke et al., 2010). In the present study, an irradiation with UV-B stimulated the expression of *PAL* in hairy roots of tartary buckwheat, showing significant expression after the irradiation (**Figure 5B**). Box-P was also conserved in the promoter region of other phenylpropanoid-biosynthesis genes and a UV responsible gene. For example, AtMYB4 in *Arabidopsis* represses the expression of the *C4H* gene, whereas UV irradiation canceled this repression and induced the expression of *C4H* (Jin et al., 2000). UV-B irradiation could trigger flavonol and anthocyanin biosynthesis in grapevine berries, which proved by up-regulated of key biosynthetic genes (*FLS1* and *UFGT*) and an increased anthocyanin concentration (Martinez-Lüscher et al., 2014). Those studies have provided useful evidence to prove the effect of UV-B exposure on the flavonoid biosynthesis.

This study demonstrated that UV-B as a major component changed dramatically in the transcript abundance of *FtpAL*, *FtCHI*, *FtCHS*, *FtF3H*, and *FtFLS-1* in *F. tataricum* hairy roots. Furthermore, *FtF3*′*H-1*, *FtF3*′*H-2*, and *FtFLS-2* genes were enhanced slightly after the UV-B treatment. A single irradiation with UV-B increased the production of rutin and quercetin for a maximum production correlated to the flavonoid biosynthesis enzyme gene expression. Through comparing with previous work, Thwe et al. (2013) investigated the various genes in the phenylpropanoid biosynthetic pathway to analyzed *in vitro* production of anthocyanin and phenolic compounds from hairy root cultures derived from two cultivars of tartary buckwheat. The result showed that phenylpropanoid biosynthetic pathway genes had expression in hairy roots, and the rutin and anthocyanin in hairy root of tartary buckwheat were identified. Thwe’s work provides useful information on the molecular and physiological dynamic process that was correlated with phenylpropanoid biosynthetic gene expression and phenolic compound content in *F. tatarium* species. Base on those works, *F. tataricum* could be achieved through a complex regulation of genes involved in the flavonoid biosynthetic pathway.

## Conclusion

We have established an efficient protocol for *A. rhizogenes-*mediated genetic transformation of tartary buckwheat (*F. tataricum*), and applied PCR analysis in the detection of hairy roots. Hairy roots cultured in 1/2 MS liquid medium supplemented with 30 g l^-1^ sucrose grew faster than normal roots under standard liquid culture conditions, and had higher rutin content. We also evaluated the effects of UV-B radiation on hairy roots and all other plant organs. We found that the rutin and quercetin content was significantly higher in hairy roots than in WT (non-transformed) roots. The expression of flavonoid biosynthetic genes was examined through quantitative real time PCR with UV-B treatment. The results showed that the expressions of key regulated genes were increased sharply in flavonoid biosynthetic pathway. Further studies on the dose-dependent UV-B irradiation could be used to determine the possible effects on tartary buckwheat flavonoids and anthocyanins incolved in the signal transduction pathway, which resulted in regulation by UV-B irradiation.

## Author Contributions

XH completed the *A. rhizogenes*-mediated transformation experiment, determination of the hairy roots rutin content and qRT-PCR experiment, JY completed effect of UV-B light stress treatment on rutin production of hairy roots experiment, YZ assisted with the *A. rhizogenes*-mediated transformation experiment, DX assisted with the rutin content determination experiment, and QZ guided the whole research as the corresponding author.

## Conflict of Interest Statement

The authors declare that the research was conducted in the absence of any commercial or financial relationships that could be construed as a potential conflict of interest.

The reviewer (Silvia Massa) and Handling Editor declared their shared affiliation, and the Handling Editor states that the process nevertheless met the standards of a fair and objective review.

## References

[B1] Abeywardena M. Y. Head R. J. (2001). Dietary polyunsaturated fatty acid and antioxidant modulation of vascular dysfunction in the spontaneously hypertensive rat. *Prostaglandins Leukot. Essent. Fatty Acids* 65 91–97. 10.1054/plef.2001.0294 11545625

[B2] Binder B. Y. K. Peebles C. A. M. Shanks J. V. San K. Y. (2009). The effects of UV-B stress on the production of terpenoid indole alkaloids in *Catharanthus roseus* hairy roots. *Biotechnol. Prog.* 25 861–865. 10.1002/btpr.97 19479674

[B3] Broeckling C. D. Huhman D. V. Farag M. A. Smith J. T. May G. D. Mendes P. (2005). Metabolic profiling of *Medicago truncatula* cell cultures reveals the effects of biotic and abiotic elicitors on metabolism. *J. Exp. Bot.* 56 323–336. 10.1093/jxb/eri058 15596476

[B4] Chen H. Chen F. (2000). Effect of yeast elicitor on the secondary metabolism of Ti-transformed *Salvia miltiorrhiza* cell suspension culture. *Plant Cell Rep.* 19 710–717. 10.1007/s002999900166 30754810

[B5] Chen L. H. Zhang B. Xu Z. Q. (2008). Salt tolerance conferred by overexpression of *Arabidopsis* vacuolar Na+/H+ antiporter gene AtNHX1 in common buckwheat (*Fagopyrum esculentum*). *Transgenic Res.* 17 121–132. 10.1007/s11248-007-9085-z 17541720

[B6] Christie J. M. Jenkins G. I. (1996). Distinct UV-B and UV-A/blue light signal transduction pathways induce chalcone synthase gene expression in *Arabidopsis* cells. *Plant Cell* 8 1555–1567. 10.1105/tpc.8.9.1555 8837509 PMC161298

[B7] Crane C. Wright E. Dixon R. Wang Z. (2006). Transgenic *Medicago truncatula* plants obtained from *Agrobacterium tumefaciens*-transformed roots and *Agrobacterium rhizogenes*-transformed hairy roots. *Planta* 223 1344–1354. 10.1007/s00425-006-0268-2 16575594

[B8] Doyle J. J. Doyle J. L. (1990). Isolation of plant DNA from fresh tissue. *Focus* 12 13–15.

[B9] Eguchi K. Sato T. (2009). Differences in the ratios of cyaniding-3-O-rutinoside to total anthocyanin under UV and non-UV conditions in tartary buckwheat (*Fagopyrum tataricum* Garten.). *Plant Prod. Sci.* 12 150–155. 10.1626/pps.12.150

[B10] Fabjan N. Rode J. Kosir I. J. Wang Z. H. Zhang Z. Kreft A. I. (2003). Tartary buckwheat (*Fagopyrum tataricum* Gaertn.) as a source of dietary rutin and quercitrin. *J. Agric. Food Chem.* 51 6452–6455. 10.1021/jf034543e 14558761

[B11] Fowler Z. L. Koffas M. A. (2009). Biosynthesis and biotechnological production of flavanones: current state and perspectives. *Appl. Microbiol. Biotechnol.* 83 799–808. 10.1007/s00253-009-2039-z 19475406

[B12] Frohnmeyer H. Ehmann B. Kretsch T. Rocholl M. Harter K. Nagatani A. (1992). Differential usage of photoreceptors for Chalcone Synthase gene-expression during plant development. *Plant J.* 2 899–906. 10.1111/j.1365-313X.1992.00899.x

[B13] Fu C. X. Xu Y. J. Zhao D. X. Ma F. S. (2006). A comparison between hairy root cultures and wild plants of *Saussurea involucrata* in phenylpropanoid production. *Plant Cell Rep.* 24 750–754. 10.1007/s00299-005-0049-6 16136313

[B14] Georgiev M. I. Pavlov A. I. Bley T. (2007). Hairy root type plant in vitro systems as sources of bioactive substances. *Appl. Microbiol. Biotechnol.* 74 1175–1185. 10.1007/s00253-007-0856-5 17294182

[B15] Guillon S. H. Tremouillaux-Guiller J. Pati P. K. Rideau M. Gantet P. (2006a). Hairy root research: recent scenario and exciting prospects. *Curr. Opin. Plant Biol.* 9 341–346. 10.1016/j.pbi.2006.03.008 16616871

[B16] Guillon S. H. Tremouillaux-Guiller J. Pati P. K. Rideau M. Gantet P. (2006b). Harnessing the potential of hairy roots: dawn of a new era. *Trends Biotechnol.* 24 403–409. 10.1016/j.tibtech.2006.07.002 16870285

[B17] Hahlbrock K. Bednarek P. Ciolkowski I. Hamberger B. Heise A. Liedgens H. (2003). Non– self recognition, transcriptional reprogramming, and secondary metabolite accumulation during plant/pathogen interactions. *Proc. Natl. Acad. Sci. U.S.A.* 100 14569–14576. 10.1073/pnas.0831246100 12704242 PMC304120

[B18] Hamill J. D. Parr A. J. Rhodes M. J. C. Robins R. J. Walton N. (1987). New routes to plant secondary products. *Nat. Biotechnol.* 5 800–804. 10.1038/nbt0887-800

[B19] Hao G. Du X. Zhao F. Shi R. Wang J. (2009). Role of nitruc oxide in UV-B-induced activation of PAL and stimulation of flavonoid biosynthesis in *Ginkgo biloba* callus. *Plant Cell Tissue Organ Cult.* 97 175–185. 10.1007/s11240-009-9513-2

[B20] Holasova M. Fidlerova V. Smrcinova H. Orsak M. Lachman J. Vavreinova S. (2001). Buckwheat– the source of antioxidant activity in functional foods. *Food Res. Int.* 35 207–211. 10.1016/S0963-9969(01)00185-5

[B21] Ionkova I. (2009). Effect of methyl jasmonate on production of ariltetralin lignans in hairy root cultures of *Linum tauricum*. *Pharmacognosy Res.* 3 102–105.

[B22] Jiang P. Burczynski F. Campbel C. Pierce G. Austria J. A. Briggs C. J. (2007). Rutin and flavonoid contents in three buckwheat species *Fagopyrum esculentum*, *F. tataricum*, and *F. homotropicum* and their protective effects against lipid peroxidation. *Food Res. Int.* 40 356–364. 10.1016/j.foodres.2006.10.009

[B23] Jin H. Cominelli E. Bailey P. Parr A. Mehrtens F. Jones J. (2000). Transcriptional repression by AtMYB4 controls production of UV-protecting sunscreens in *Arabidopsis*. *EMBO J.* 19 6150–6161. 10.1093/emboj/19.22.6150 11080161 PMC305818

[B24] Kaneko M. Hwang E. I. Ohnishi Y. Horinouchi S. (2003). Heterologous production of flavanones in *Escherichia coli*: potential for combinatorial biosynthesis of flavonoids in bacteria. *J. Ind. Microbiol. Biotechnol.* 30 456–461. 10.1007/s10295-003-0061-1 12759810

[B25] Kim K. H. Lee Y. H. Kim D. Park Y. H. Lee J. Y. Hwang Y. S. (2004). *Agrobacterium*-mediated genetic transformation of *Perilla frutescens*. *Plant Cell Rep.* 23 386–390. 10.1007/s00299-004-0825-8 15368075

[B26] Kim Y. K. Li X. Xu H. Park N. I. Uddin M. R. Pyon J. Y. (2009). Production of phenolic compounds in hairy root culture of tartary buckwheat (*Fagopyrum tataricum* Gaertn). *J. Crop Sci. Biotechnol.* 12 53–58. 10.1371/journal.pone.0065349

[B27] Kim Y. K. Xu H. Park W. T. Park N. I. Lee S. Y. Park S. U. (2010). Genetic transformation of buckwheat (*Fagopyrum esculentum* M.) with *Agrobacterium rhizogenes* and production of rutin in transformed root cultures. *Aust. J. Crop Sci.* 4 485–490.

[B28] Kliebenstein D. J. Lim J. E. Landry L. G. Last R. L. (2002). *Arabidopsis* UVR8 regulates ultraviolet-B signal transduction and tolerance and contains sequence similarity to human Regulator of chromatin condensation 1. *Plant Physiol.* 130 234–243. 10.1104/pp.005041 12226503 PMC166556

[B29] Kreft S. Strukelj B. Gaberscik A. Kreft I. (2002). Rutin in buckwheat herbs grown at different UV-B radiation levels: comparison of two UV spectrophotometric and an HPLC method. *J. Exp. Bot.* 53 1801–1804. 10.1093/jxb/erf032 12147730

[B30] Krkošková B. Mrázová Z. (2005). Prophylactic components of buckwheat. *Food Res. Int.* 38 561–568. 10.1016/j.foodres.2004.11.009

[B31] Kubasek W. L. Shirley B. W. McKillop A. Goodman H. M. Briggs W. Ausubel F. M. (1992). Regulation of flavonoid biosynthetic genes in germinating *Arabidopsis* seedlings. *Plant Cell* 4 1229–1236. 10.2307/3869409 12297632 PMC160210

[B32] Lee H. M. Yoon E. S. Jeong J. H. Choi Y. E. (2004). *Agrobacterium rhizogenes*-mediated transformation of *Taraxacum platycarpum* and changes of morphological characters. *Plant Cell Rep.* 22 822–827. 10.1007/s00299-004-0763-5 14986056

[B33] Lee S. Y. Cho S. I. Park M. H. Kim Y. K. Choi J. E. Park S. U. (2007). Growth and rutin production in hairy root cultures of buckwheat (*Fagopyrum esculentum* M.). *Prep. Biochem. Biotechnol.* 37 239–246. 10.1080/10826060701386729 17516253

[B34] Li X. Park N. I. Xu H. Woo S. H. Park C. H. Park S. U. (2010). Differential expression of flavonoid biosynthesis genes and accumulation of phenolic compounds in common buckwheat (*Fagopyrum esculentum*). *J. Agric. Food Chem.* 58 12176–12181. 10.1021/jf103310g 21062042

[B35] Martinez-Lüscher J. Sánchez-Díaz M. Delrot S. Aguirreolea J. Pascual I. Gomès E. (2014). Ultraviolet-B radiation and water deficit interact to alter flavonol and anthocyanin profiles in grapevine berries through transcriptomic regulation. *Plant Cell Physiol.* 55 1925–1936. 10.1093/pcp/pcu121 25231967

[B36] Murashige T. Skoog F. (1962). A revised medium for rapid growth and bioassays with tobacco tissue culture. *Physiol. Plant.* 15 473–497. 10.1111/j.1399-3054.1962.tb08052.x

[B37] Ožbolt L. Kreft S. Kreft I. Germ M. Stibilj V. (2008). Distribution of selenium and phenolics in buckwheat plants grown from seeds soaked in Se solution and under different levels of UV-B radiation. *Food Chem.* 110 691–696. 10.1016/j.foodchem.2008.02.073

[B38] Pandey R. P. Sohng J. K. (2013). “Genetics of flavonoids,” in *Natural Products*, eds Ramawat K. G. Mérillon J.-M. (Heidelberg: Springer Berlin), 1617–1645.

[B39] Park B. J. Park J. I. Chang K. J. Park C. H. (2004). “Comparison in rutin content in seed and plant of tartary buckwheat (*Fagopyrum tataricum*),” in *Proceedings of the 9th International Symposium on Buckwheat*, Prague, 626–629.

[B40] Park C. H. Kim Y. B. Choi Y. S. Heo K. Kim S. L. Lee K. C. (2000). Rutin content in food products processed from groats, leaves and flowers of buckwheat. *Fagopyrum* 17 63–66.

[B41] Park N. I. Li X. Uddin M. R. Park S. U. (2011). Phenolic compound production by different morphological phenotypes in hairy root cultures of *Fagopyrum tataricum* Gaertn. *Arch. Biol. Sci.* 63 193–198. 10.2298/ABS1101193P

[B42] Petit A. David C. Dahl G. A. Ellis J. G. Guyon P. Casse-Delbart F. (1983). Further extension of the opine concept: plasmids in *Agrobacterium rhizogenes* cooperate for opine degradation. *Mol. Genet. Genomics* 190 204–214. 10.1007/BF00330641

[B43] Prakash G. Srivastava A. K. (2008). Statistical elicitor optimization studies for the enhancement of azadirachtin production in bioreactor *Azadirachta indica* cell cultivation. *Biochem. Eng. J.* 40 218–226. 10.1016/j.bej.2007.12.017

[B44] Qin P. Y. Wu L. Yao Y. Ren G. X. (2013). Changes in phytochemical compositions, antioxidant and α-glucosidase inhibitory activities during the processing of tartary buckwheat tea. *Food Res. Int.* 50 562–567.

[B45] Rao S. R. Ravishankar G. A. (2002). Plant cell cultures: chemical factories of secondary metabolites. *Biotechnol. Adv.* 20 101–153. 10.1016/S0734-9750(02)00007-1 14538059

[B46] Rizzini L. Favory J.-J. Cloix C. Faggionato D. O’Hara A. Kaiserli E. (2011). Perception of UV-B by the *Arabidopsis* UVR8 protein. *Science* 332 103–106. 10.1126/science.1200660 21454788

[B47] Santos C. N. S. Koffas M. A. Stephanopoulos G. (2011). Optimization of a heterologous pathway for the production of flavonoids from glucose. *Metab. Eng.* 13 392–400. 10.1016/j.ymben.2011.02.002 21320631

[B48] Smetanska I. (2008). Production of secondary metabolites using plant cell cultures. *Adv. Biochem. Eng. Biotechnol.* 111 187–228. 10.1007/10_2008_103 18594786

[B49] Stracke R. Favory J. J. Gruber H. Bartelniewoehner L. Bartels S. Binkert M. (2010). The *Arabidopsis* bZIP transcription factor HY5 regulates expression of the PFG1/MYB12 gene in response to light and ultraviolet-B radiation. *Plant Cell Environ.* 33 88–103. 10.1111/j.1365-3040.2009.02061.x 19895401

[B50] Thiruvengadam M. Praveen N. Kim E. Kim S. Chung I. (2014). Production of anthraquinones, phenolic compounds and biological activities from hairy root cultures of *Polygonum multiflorum* Thunb. *Protoplasma* 251 555–566. 10.1007/s00709-013-0554-3 24091894

[B51] Thwe A. A. Kim J. K. Li X. H. Kim Y. B. Uddin M. R. Kim S. J. (2013). Metabolomic analysis and phenylpropanoid biosynthesis in hairy root culture of tartary buckwheat cultivars. *PLoS ONE* 8:e65349 10.1371/journal.pone.0065349 PMC3683005 23799007

[B52] Timotijevic G. S. Milisavljevic M. D. Radovic S. R. Konstantinovic M. M. Maksimovic V. R. (2010). Ubiquitous aspartic proteinase as an actor in the stress response in buckwheat. *J. Plant Physiol.* 167 61–68. 10.1016/j.jplph.2009.06.017 19643510

[B53] Tomotake H. Yamamoto N. Kitabayashi H. Kawakami A. Kayashita J. Ohina H. (2007). Preparation of tartary buckwheat protein product and its improving effect on cholesterol metabolism in rats and mice fed cholesterol-enriched diet. *J. Food Sci.* 72 528–533. 10.1111/j.1750-3841.2007.00474.x 17995668

[B54] Tsurunaga Y. Takahashi T. Katsube T. Kudo A. Kuramitsu O. Ishiwata M. (2013). Effect of UV-B irradiation on the levels of anthocyanin, rutin and radical scavenging activity of buckwheat sprouts. *Food Chem.* 141 552–556. 10.1016/j.foodchem.2013.03.032 23768393

[B55] Van Larebeke N. Genetello C. H. Hernalsteens J. P. De Picker A. Zaenen I. Messens E. (1977). Transfer of Ti plasmids between *Agrobacterium* strains by mobilization with the conjugative plasmid RP4. *Mol. Genet. Genomics* 152 1119–1124.

[B56] Watanabe M. (1998). Catechins as antioxidants from buckwheat (*Fagopyrum esculentum* Moench) groats. *J. Agric. Food Chem.* 46 839–845. 10.1021/jf9707546

[B57] Winkel-Shirley B. (2001). Flavonoid biosynthesis, a colorful model for genetics, biochemistry, cell biology, and biotechnology. *Plant Physiol.* 126 485–493. 10.1104/pp.126.2.485 11402179 PMC1540115

[B58] Wojcicki J. Barcew-Wiszniewska B. Samochowiec L. Rozewicka L. (1995). Extractum Fagopyri reduces atherosclerosis in high-fat diet fed rabbits. *Pharmazie* 50 560–562. 7568319

[B59] Yang D. C. Choi Y. E. (2000). Production of transgenic plants via *Agrobacterium rhizogenes*-mediated transformation of *Panax ginseng*. *Plant Cell Rep.* 19 491–496. 10.1007/s002990050761 30754888

[B60] Yang N. Ren G. X. (2008). Determination of D -chiro-Inositol in tartary buckwheat using high-performance liquid chromatography with an evaporative light-scattering detector. *J. Agric. Food Chem.* 56 757–760. 10.1021/jf0717541 18167075

[B61] Yao Y. Shan F. Bian J. S. Chen F. Wang M. F. Ren G. X. (2008). D- chiro-Inositol-enriched tartary buckwheat bran extract lowers the blood glucose level in KK-Aymice. *J. Agric. Food Chem.* 56 10027–10031. 10.1021/jf801879m 18921966

[B62] Yu S. Kwok K. H. Doran P. M. (1996). Effect of sucrose, exogenous product concentration, and other culture conditions on growth and steroidal alkaloid production by *Solanum* avidare hairy roots. *Enzyme Microb. Technol.* 18 238–243. 10.1016/0141-0229(95)00057-7

[B63] Zhao J. Zhou L. Wu J. (2010). Effects of biotic and abiotic elicitors on cell growth and tanshinone accumulation in *Salvia miltiorrhiza* cell cultures. *Appl. Microbiol. Biotechnol.* 87 137–144. 10.1007/s00253-010-2443-4 20195862

[B64] Zhao J. Zou L. Zhang C. Li Y. Peng L. Xiang D. (2014a). Efficient production of flavonoid in *Fagopyrum tartaricum* hairy root cultures with yeast polysaccharide elicitation and medium renewal process. *Pharmacogn. Mag.* 10 234–240. 10.4103/0973-1296.137362 25210309 PMC4159915

[B65] Zhao J. Xiang D. Peng L. Liang Z. Wang Y. Zhao G. (2014b). Enhancement of rutin production in *Fagopyrum tataricum* hairy root cultures with its endophytic fungal elicitors. *Prep. Biochem. Biotechnol.* 44 782–794. 10.1080/10826068.2013.867872 24279735

