# Supplementary material for: Efficient Rutin and Quercetin Biosynthesis through Flavonoids-Related Gene Expression in Fagopyrum tataricum Gaertn. Hairy Root Cultures with UV-B Irradiation
Source: Front Plant Sci. 2016 Feb 4;7:63. doi: 10.3389/fpls.2016.00063 (PMC4740399; doi:10.3389/fpls.2016.00063)

Supplementary Material:

S1. The HPLC chromatogram and standard curve of rutin standard sample.

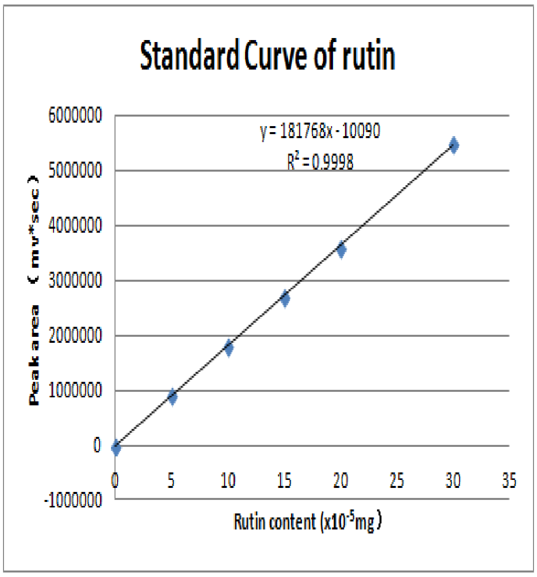

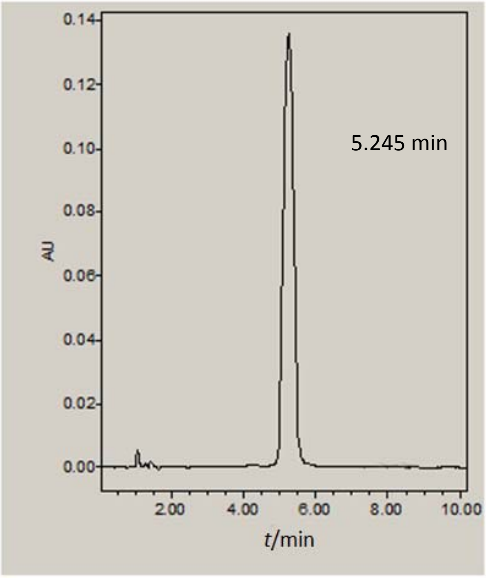

S2. The HPLC chromatogram and standard curve of quercetin standard sample.

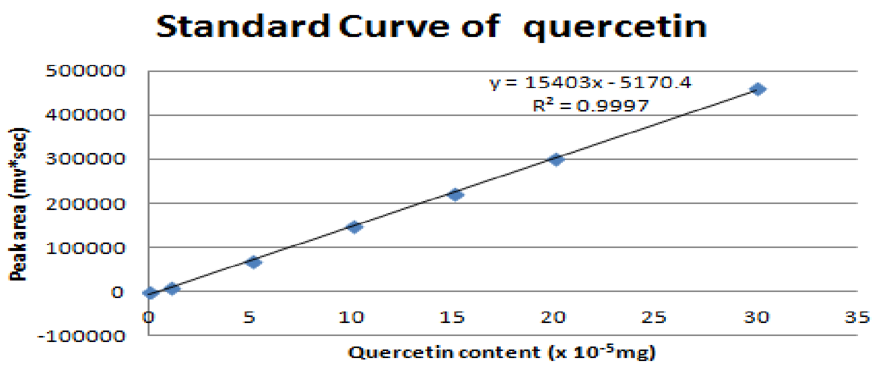

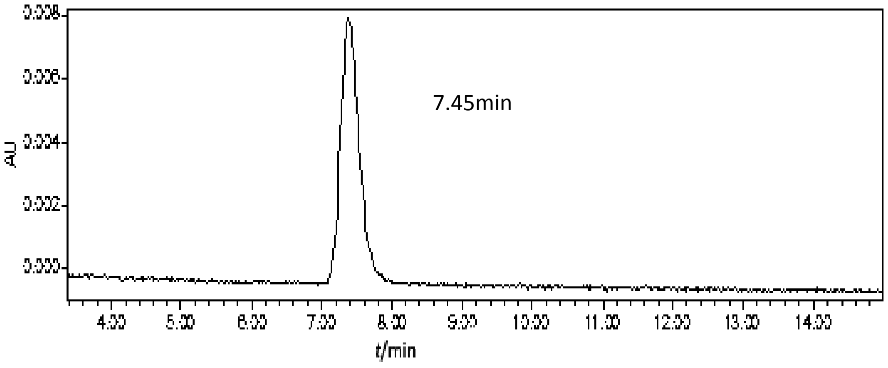

S3. The HPLC chromatogram of (A) rutin and (B) quercetin in hairy root line TB7 in 370nm. The retention time of rutin and quercetin was 5.27 min and 7.50 min, respectively.

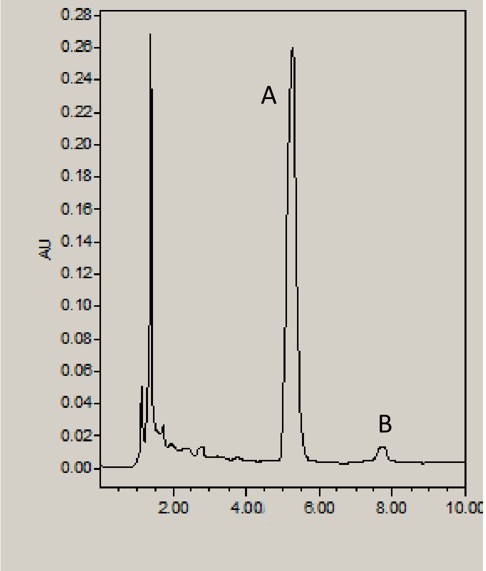

S4. PCR analysis for the presence of *rolB*, *aux1* and *virD* genes in hairy root lines of *F. tataricum*. transformed by *A. rhizogenes* strain 15834. Lane M, marker. Lane 1, non-transformed roots (negative control, NC). Lane 2, plasmid DNA (positive control, PC). Lanes 4-13, transformed hairy root clones.

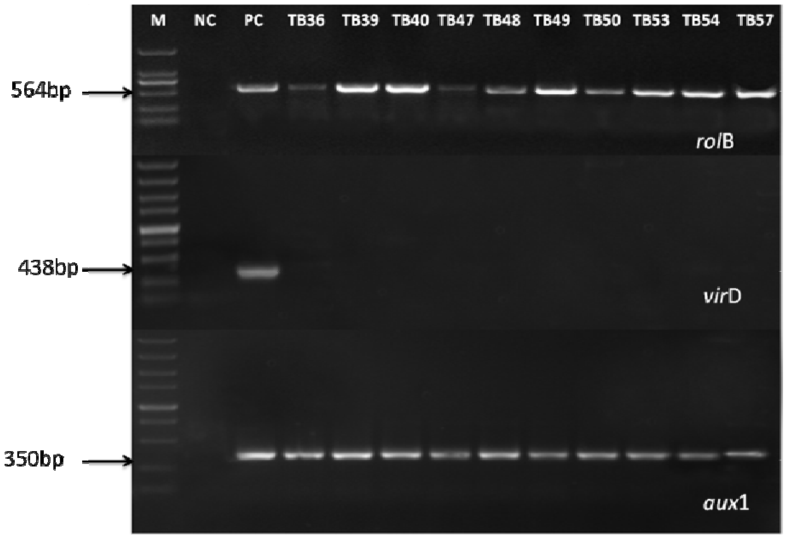

Supplement: Supplementary file 1 [file Presentation_1.PDF]
